# Reversible Mechanochromic Delayed Fluorescence in 2D Metal–Organic Micro/Nanosheets: Switching Singlet–Triplet States through Transformation between Exciplex and Excimer

**DOI:** 10.1002/advs.201801187

**Published:** 2018-09-27

**Authors:** Yongsheng Yang, Xiaogang Yang, Xiaoyu Fang, Ke‐Zhi Wang, Dongpeng Yan

**Affiliations:** ^1^ Beijing Key Laboratory of Energy Conversion and Storage Materials College of Chemistry Beijing Normal University Beijing 100875 P. R. China; ^2^ Institute of Catalysis for Energy and Environment College of Chemistry and Chemical Engineering Shenyang Normal University Shenyang 110034 P. R. China; ^3^ State Key Laboratory of Chemical Resource Engineering Beijing University of Chemical Technology Beijing 100029 P. R. China

**Keywords:** excimers, exciplexes, mechanochromic delayed fluorescence, metal–organic microsheets, singlet–triplet states

## Abstract

Mechanochromic luminescent materials have attracted much attention and present a variety of applications in information security, data recording, and storage devices. However, most of these smart luminescent systems are based on typical fluorescence and/or phosphorescence mechanisms; the mechanochromic delayed fluorescence (MCDF) materials involving switching singlet and triplet states are rarely studied to date. Herein, new 2D layered metal–organic micro/nanosheets, [Cd(9‐AC)_2_(BIM)_2_] (named as MCDF‐1; 9‐AC = anthracene‐9‐carboxylate and BIM = benzimidazole) and its solvate form containing interlayer CH_3_CN (named as MCDF‐2), which exhibit reversible mechanochromic delayed fluorescence characteristics, are presented. With applying the mechanical force, the luminescent center of MCDF‐1 can be converted from 9‐AC/BIM exciplex to 9‐AC/9‐AC excimer, resulting in alternations of delayed fluorescence. Such luminescent change can be further recovered by CH_3_CN fumigation, accompanied by the structural transformation from MCDF‐1 to MCDF‐2. Furthermore, the force‐responsive process also refers to the energy redistribution between singlet and triplet states as inferred by both temperature‐dependent photophysics and theoretical calculations. Therefore, this work not only develops new 2D micro/nanosheets as MCDF materials, but also supplies a singlet–triplet energy switching mechanism on their reversible mechanochromic process.

Smart luminescent materials which respond to the external stimuli (such as mechanical, vaporous, electric, and thermal conditions) have received increasing attention in various fields of security systems, data recording, and storage devices.^1^ Mechanochromic luminescent materials are characterized as the changes of emission wavelength, intensity, and/or lifetime upon mechanical shearing, grinding, and rubbing of the solid‐state systems. This process is usually due to the alternation of intermolecular interactions, such as π–π stacking and hydrogen bonds.^2^ The subsequent reversion to the original state is usually achieved upon vaporous or thermal treatment. Therefore, rational balance of different intermolecular forces becomes the key to the design of current mechano‐responsive luminescent materials. Although the study of pure organic illuminants (including organic dyes, liquid crystals, and polymers) with mechanochromism properties has achieved great progress, it is still highly desirable and challenging to develop new mechanisms toward mechanochromic systems.^3^ During last few years, metal–organic hybrid materials (such as coordination polymers, metal–organic frameworks and complexes) with mechanochromism have also been increasingly investigated. According to the mechanisms, metal–organic mechanochromic materials can be divided into two types: (1) most of as‐reported systems are metal‐centered phosphorescence emission based on altering the arrangement of metals or clusters under the mechanical stimulus, such as Au^I^,^4^ Pt^II^,^5^ Cu^I^,^6^ and Ir^III^,^7^ etc.; (2) the others involve fluorescence emission based on the change of the stacking mode and interaction of ligands, such as ligand‐based Zn^II^ 8 and Cd^II^.^9^

In recent years, delayed fluorescent (DF) materials, particularly for thermally activated delayed fluorescence (TADF), have made significant progress in the field of light‐emitting diodes (LEDs).^10^ Compared with the typical fluorescence and phosphorescence processes, DF could achieve high emission intensity and long‐lived emission lifetimes simultaneously, which is beneficial to the improvement of luminescence efficiency in illumination and security fields.^11^ However, to the best of our knowledge, no report has focused on the mechanochromic delayed fluorescent (MCDF) materials based on metal–organic hybrid system. This is probably due to the fact that tuning of singlet–triplet energy distribution through mechanical force is still difficult. Recently, Graves et al. reported an exciplex blended film formed by two aromatic conjugated molecules achieved a smaller singlet–triplet energy splitting and DF property.^12^ Therefore, construction of exciplex components in metal–organic hybrid materials may serve as a promising means to obtain DF. In this contribution, we designed and synthesized novel 2D layered Cd(II)‐based metal–organic hybrid and its solvate form (containing CH_3_CN), which exhibit interesting MCDF properties.

During last few years, the 2D layered structures have received much attention due to the unique properties and applications in the fields of opto/electronic transistors,^13^ LEDs,^14^ (electro)chemical sensors,^15^ solar cells,^16^ and plasmon–photonic polaritons.^17^ In theory, the weak interactions between 2D stacked monolayers could facilitate the slipping and deformation of molecular sheets under the external force, which favors the adjustment of arrangement and interactions between chromophores toward force‐responsive characteristics.^18^ We chose anthracene‐9‐carboxylic acid (9‐HAC) and benzimidazole (BIM) as coordination ligands based on the following expectation: first, the conjugated π‐systems of anthracene ring is of interest in the triplet exciton,^19^ and the carbonyl group can also enhance spin–orbit coupling between singlet and triplet states;^20^ second, BIM is able to dimerize with anthracene‐9‐carboxylate (9‐AC) through hydrogen bond, π–π, and C—H⋅⋅⋅π stacking interactions, and the formation of excimer or exciplex between two ligands hold the promise for potential MCDF performance. Furthermore, the selection of heavy atom Cd as the metal unit is due to its benefit on mixing singlet and triplet states of different electronic configurations and promoting energy transfer between different states, which enhances the possibility of reverse intersystem crossing. The as‐prepared MCDF‐1 and MCDF‐2 materials feature a reversible DF response under the mechanical and vaporous stimuli (**Scheme** **1**). This characteristic is realized by the transform between exciplex (BIM/9‐AC) and excimer (9‐AC/9‐AC) as a result of the change in weak intermolecular forces and stacking interactions under external grinding. Meanwhile, this process also achieves the change in the energy distribution between singlet and triplet states.^21^ Therefore, these findings provide a new platform to construct 2D MCDF micro/nanostructures based on a transformation mechanism between exciplex and excimer states.

**Scheme 1:**
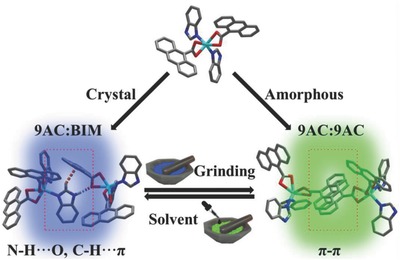
Schematic representations of the MCDF‐1 with mechanochromic and vapochromic properties.

[Cd(9‐AC)_2_(BIM)_2_] (MCDF‐1) and its solvate form (MCDF‐2) are assembled from Cd(NO)_2_·4H_2_O, 9‐HAC and BIM by a hydrothermal method under 150 °C (Table S1, Supporting Information). In both MCDF‐1 and MCDF‐2, each node CdO_4_N_2_ polyhedron is formed by two 9‐AC chelating ligands and two coordinated BIM ligands (Figure S1a,b, Supporting Information); these polyhedrons are connected by both hydrogen bonding (1.921 and 1.907 Å) and the C—H⋅⋅⋅π (2.849 and 2.804 Å) interactions between BIM and 9‐AC in adjacent units to form 2D monolayers (Figure S1c,d, Supporting Information), which further stack in an AB packing pattern (**Figure** **1**). For MCDF‐2, the CH_3_CN molecules intercalate between the adjacent monolayers by the hydrogen bond (C—H⋅⋅⋅O) which reduce the degree of layer dislocation and slightly increase the interlayer distance from 10.81 to 11.35 Ǻ. As shown in Figure S2 (Supporting Information), good agreement exists between the simulated and experimental powder X‐ray diffraction (PXRD) patterns, verifying the high purity of the as‐synthesized MCDF‐1 and MCDF‐2 samples. The difference in composition between MCDF‐1 and MCDF‐2 can also be observed by the Fourier transform infrared (FTIR) spectra (Figure S3, Supporting Information): the characteristic absorption peaks of the stretching vibration of C≡N (2334 and 2377 cm^−1^) can be found in MCDF‐2, which indicates the presence of CH_3_CN.

**Figure 1:**
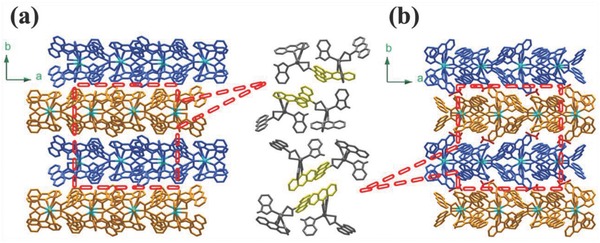
a,b) The AB packing patterns of the MCDF‐1 and MCDF‐2. The *ac* planes are highlighted in blue and orange to better illustrate the layered structure (the middle schemes are the relative position of intralayer 9‐AC ligands).

Both of MCDF‐1 and MCDF‐2 present 2D sheet‐like crystals. Taking MCDF‐1 as an example, scanning electron microscopy (SEM) shows that the cross‐sectional morphology (**Figure** **2**a) is characterized as multiple‐layered stacking fashion, in which 2D layers are oriented normal to the stacking direction, suggesting the formation of 2D metal–organic micro/nanosheets. Upon ultrasonic treatment in water, the multiple‐layered stacking can be deaggregated into monodispersed disk‐like nanostructures (Figure 2b). The atomic force microscopy (AFM, Figure 2c) shows that the thickness of the 2D nanodisks is around 9 nm, with the size of ≈500 nm, indicating that the ultrathin nanodisk structures correspond to stacking of eight monolayers within the metal–organic hybrid. If the MCDF‐1 nanodisks underwent a grinding treatment, irregular elliptical‐like micro/nanosheets can be obtained (Figure 2d), which is regarded as the slippage and compression of the interlayers. The above phenomena illustrate that the interaction between 2D layers in the pristine MCDF‐1 material is relatively weak, and thus the position and staking fashion between adjacent monolayers can be easily alternated upon grinding treatment.

**Figure 2:**
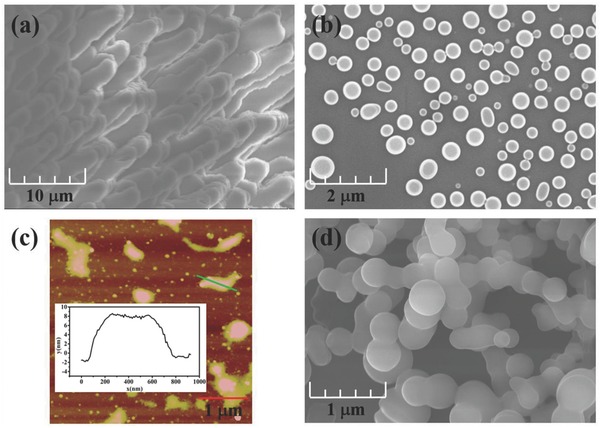
a) SEM image of cross‐sectional morphology of MCDF‐1 crystal sample. b) SEM image of MCDF‐1 disk‐like micro/nanosheets obtained by ultrasonic treatment in a deionized water solvent. c) AFM image of MCDF‐1 nanosheets obtained by ultrasonic treatment. d) SEM image of MCDF‐1 elliptical‐like micro/nanosheets obtained by ultrasonic and grinding treatment.

To study DF properties, both of the prompt (solid line) and the delay‐detected photoluminescence (PL) spectra (dashed line, 10 µs) for two solid‐state micro/nanosheets were measured at room temperature (**Figure** **3**a). The difference between prompt and delayed fluorescence spectra can be related to the coordination component (CdO_4_N_2_) emission center with a longer luminescence lifetime, and thus affects the final peak width of the delay‐detected photoluminescence spectra.^22^ The MCDF‐1 and MCDF‐2 exhibit blue fluorescence with single broad emission bands centered at 460 and 470 nm, respectively (excitation wavelength: 340 nm, Figure S4, Supporting Information). Compared with the pristine 9‐AC monomer (emission at 410 nm),^23^ the redshifted luminescence for both MCDF‐1 and MCDF‐2 can be attributed to exciplex emission from a stacked 9‐AC/BIM dimer, as also confirmed by their crystal structures. Additionally, an emissive redshift of 10 nm for MCDF‐2 relative to MCDF‐1 is consistent with the fact that the interlayer slipping occurs for MCDF‐2, which leads to the partial aggregation of 9‐AC ligands as observed in the stacking structure (Figure 1). To detect the excitation states of 2D metal–organic micro/nanosheets, the time‐resolved photoluminescence decay spectra were measured, and their corresponding fluorescence lifetimes in the solid state were determined as 345.2 and 324.7 µs at room temperature, respectively (Figure 3b and Figure S5, Supporting Information). Such long‐lived blue DF is consistent with the formation of 9‐AC‐based exciplex in the crystalline samples.

**Figure 3:**
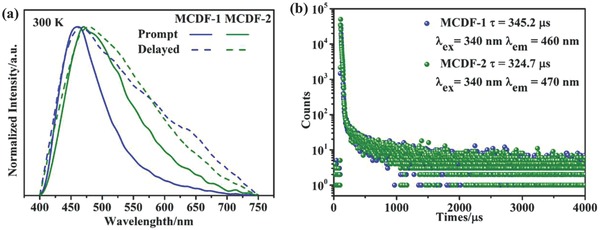
a) Normalized prompt (solid line) and delay‐detected photoluminescence spectra (dashed line, 10 µs) of MCDF‐1 and MCDF‐2, respectively. b) Time‐resolved emission decay curves (the excitation wavelength is 340 nm and the emission wavelengths are 460 and 470 nm, respectively) for MCDF‐1 and MCDF‐2 under ambient conditions.

MCDF‐1 was selected to study the mechanochromic delayed luminescence behavior (**Figure** **4**a). For the pristine MCDF‐1 (named as MCDF‐1B), the solid‐state sample exhibits blue luminescence centered at 460 nm. After grinding in a mortar, the luminescence presents a redshift emission toward 490 nm, resulting in a green‐emitting powder (named as MCDF‐1G) that can be easily observed by the naked eye. Interestingly, solvent CH_3_CN fuming on MCDF‐1G powder results in a recovery of the emission at 470 nm, as the same position with MCDF‐2. Therefore, MCDF‐1 undergoes a spectral change between blue (460 or 470 nm) and green (490 nm) color upon alternative treatment by grinding and fuming (Figure 4b); such reversible process can be recycled for at least three times (Figure 4c). To further study the influence of grinding on the crystal structure and photophysical properties of MCDF‐1, PXRD (Figure 4d) and solid‐state UV–vis spectra (Figure 4e) have been measured at room temperature before and after grinding. The PXRD pattern of MCDF‐1B shows an intense and sharp diffraction signal, confirming highly crystalline phase with ordered stacking. By contrast, MCDF‐1G shows very weak and broad reflection peaks, which suggests a quasi‐amorphous state: the total absence of the (020) diffraction illustrates that the layered long‐range order vertical to the micro/nanosheets has been destroyed, while the remaining (004) diffraction suggests that the 2D order within the monolayer has partially maintained during the grinding process. The crystalline‐to‐amorphous transformation for solid‐state MCDF‐1 is also in agreement with the morphological changes for both the 2D nanodisk structures (Figure 2b,d) and the macrosized bulk crystal before and after grinding (Figure S6a,b, Supporting Information), in which the long‐range stacking characteristic has been destroyed upon force stimulus.

**Figure 4:**
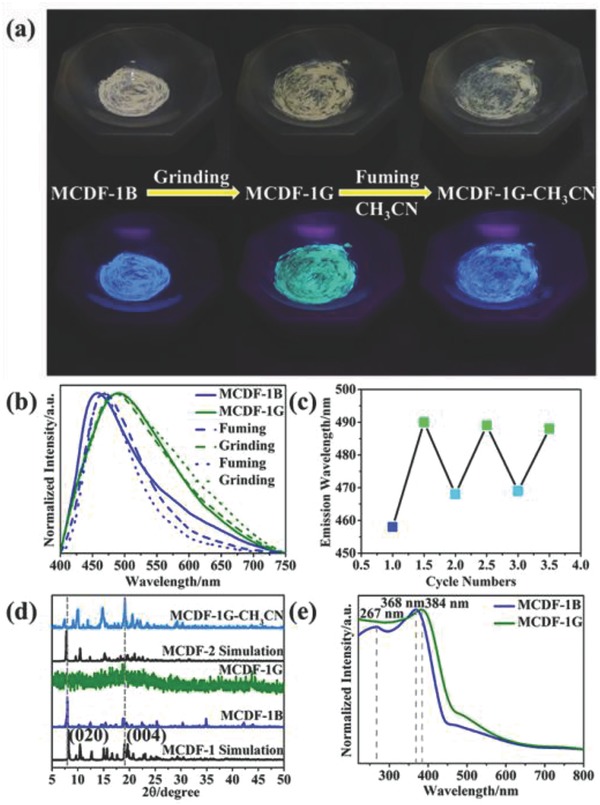
a) Solid‐state emission colors of MCDF‐1 upon grinding and fuming by CH_3_CN under ambient light and UV lamp (irradiated at 365 nm). b) Reversible variation of the emission spectra for MCDF‐1 under alternate treatment of grinding and fuming by CH_3_CN. c) Reversible variation of the maximum emission intensity (460 or 470 and 490 nm) for MCDF‐1 under alternate treatment of grinding and fuming by CH_3_CN. d) Powder X‐ray diffraction patterns and e) solid‐state UV–vis spectra of the MCDF‐1.

After fuming the MCDF‐1G with CH_3_CN vapor, the XRD peaks of the sample (named as MCDF‐1G‐CH_3_CN) reappear, which indicates that the amorphous state retunes back to the orderly aggregation by molecular repacking. Interestingly, the PXRD pattern of recovered structure matches well with that of MCDF‐2, suggesting that the CH_3_CN molecules could serve as a template to drive the self‐assembly of the 2D layered structures, and the restacking of 2D structure can be observed in the recovered multiple‐layered morphology after CH_3_CN treatment (Figure S6c, Supporting Information). The appearance of restacked MCDF‐2 phase is also in agreement with the emission at 470 nm. These observation further shows that through the consecutive friction and fumigation process, the crystal‐to‐crystal transformation between MCDF‐1 to MCDF‐2 can be achieved (Figure S7, Supporting Information). Moreover, the UV absorption band of MCDF‐1G has a slight redshift compared to MCDF‐1B, which is consistent with the redshift of the emission spectra. Notably, the green emission of MCDF‐1G at 490 nm is very close to the intrinsic luminescence of solid‐state 9‐HAC ligand (Figure S8, Supporting Information). It has been known that the redshift of the emission of solid‐state 9‐HAC, relative to its monomer state (410 nm), is ascribed to excimer formation of 9‐HAC dimer. As well, the enhancement of the stacking between 9‐AC molecules results in the redshift of MCDF‐2 relative to MCDF‐1. Therefore, it can be speculated that grinding‐induced redshift emission of MCDF‐1 can be attributed to the transformation of intermolecular interactions from 9‐AC/BIM exciplex to 9‐AC/9‐AC excimer, since the interaction between 9‐AC can be further enhanced if the occurrence of slipping and dislocation between adjacent monolayers during the crystal‐to‐amorphous process (Scheme 1). Additionally, we have also tried to use other common solvents (such as methanol and ethanol) to drive the reversible behavior. It was found that these small solvent molecules that form potential hydrogen bonds with intralayer ligands can also restore the changes of color (Figures S9 and S10, Supporting Information).

To explore the impact of grinding on DF, the prompt and the delay‐detected photoluminescence spectra (10 µs) of MCDF‐1 before (MCDF‐1B) and after grinding (MCDF‐1G) were measured in the temperature range from 77 to 427 K. The temperature‐dependent prompt emission profiles of MCDF‐1B and MCDF‐1G are shown in **Figure** **5**. For MCDF‐1B, the temperature‐dependent prompt spectra show the same fluorescence band at about 460 nm (λ_ex_ = 340 nm), and the intensity is reduced systematically upon increasing temperature from 77 to 427 K, in which the decreasing trend is very weak from 77 to 287 K. Compared with the lifetime of MCDF‐1B (424.8 µs) at 77 K, the fluorescence decay maintains relatively slow (301.3 µs) at 427 K, which indicates that MCDF‐1B have a certain amount of TADF characteristic. For MCDF‐1G, upon decreasing the temperature, the emission band undergoes an obvious redshift, with the emission at 540 nm becomes the major band. Such redshifted emission at low temperature also appears for the pristine 9‐HAC solid, which is related to the decrease of vibration and rotation of anthracycline units as the temperature decreased. The similarity in their spectral change further confirms the occurrence of 9‐AC excimers in the MCDF‐1G. Interestingly, the decay rate of intensity and lifetime of MCDF‐1G were faster than that of MCDF‐1B from 77 to 427 K. This suggests that the TADF characteristic of MCDF‐1B is more pronounced than MCDF‐1G. Furthermore, the temperature‐dependent delay‐detected photoluminescence spectra (10 µs) of MCDF‐1B (Figure S11 and Table S2, Supporting Information) show slightly redshifted emission maxima (less than 9 nm) relative to the prompt results, suggesting the high‐efficiency DF performance. While for the MCDF‐1G, the delay‐detected photoluminescence spectra (10 µs) present an obvious redshift (more than 45 nm) compared to the prompt results from room temperature to 77 K, suggesting the occurrence of phosphorescent emission of the MCDF‐1G. Because reverse intersystem crossing (RISC) is highly temperature dependent, and TADF is largely suppressed at low temperature,^24^ the delay‐detected emission at 77 K is most likely attribute to triplet phosphorescent emission. Therefore, it is more convincing to estimate energy gaps Δ*E*_ST_ at 77 K condition. Based on the low‐temperature (77 K) prompt and delay‐detected photoluminescence spectra (10 µs) for the MCDF‐1B and MCDF‐1G, the energy gaps between S_1_ and T_1_ (Δ*E*_ST_) can be evaluated as ≈0.03 and 0.19 eV, respectively. Therefore, the energy level diagram of the relevant photophysical processes can be illustrated as shown in **Figure** **6**, in which the difference of DF performance between MCDF‐1B and MCDF‐1G is related to the alternation of their Δ*E*_ST_ before and after grinding, which could affect the reverse intersystem crossing process effectively. Additionally, as shown in Figure S12 (Supporting Information), the thermogravimetric analysis (TGA) curve of MCDF‐1 shows good thermal stability below 225 °C, suggesting that the structure of MCDF‐1 maintains during the temperature‐dependent emission detection.

**Figure 5:**
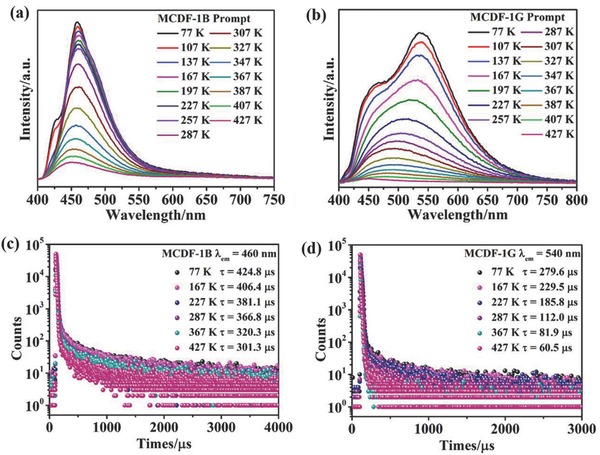
a,b) Prompt spectra of MCDF‐1B and MCDF‐1G measured at temperatures from 77 to 427 K. c) Emission decay curves of MCDF‐1B in the solid state at 460 nm with different temperatures. d) Emission decay curves of MCDF‐1G in the solid state at 540 nm with different temperatures.

**Figure 6:**
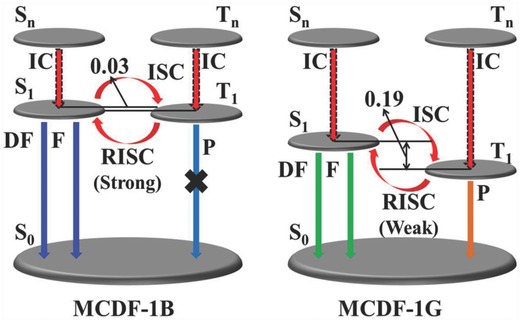
The energy level diagram of the relevant photophysical processes (fluorescence (F), phosphorescence (P), and delayed fluorescence (DF)) for MCDF‐1B and MCDF‐1G. IC: internal conversion, ISC: intersystem crossing, RISC: reversible intersystem crossing.

To better understand the mechanochromic mechanism, density functional theoretical (DFT) calculation was performed on MCDF‐1 single crystal model. As shown in Figure S13 and Table S3 (Supporting Information), frontier orbital analysis shows that the energy gap of MCDF‐1 has been further reduced relative to the pristine BIM and 9‐AC molecules, which is an important standard for the formation of exciplex. Moreover, Figure S14 (Supporting Information) shows the presence of possible transition peaks at 458.2, 536.3, and 572.5 nm, which match with the prompt emission spectra of MCDF‐1B and MCDF‐1G at 77 K. The electronic transition at 458.2 nm is populated on both of 9‐AC (HOMO‐1) and BIM ligands (LUMO+3), corresponding to 9‐AC/BIM exciplex formation (460 nm in experiment). Whereas the transitions at 536.3 and 572.5 nm are only distributed on 9‐AC ligand which can be attributed to the formation of 9‐AC/9‐AC excimer in experiment. This also explains the mechanochromic emission based on transformation from 9‐AC/BIM exciplex to 9‐AC/9‐AC excimer upon grinding. Meanwhile, total/partial electronic density of states (TDOS/PDOS) analyses (Figure S15, Supporting Information) reveal that, around the Fermi level, the TDOS mainly consists of the 2p electrons from C/O/N atoms of the BIM and 9‐AC ligands and the d electrons from the Cd atoms. These facts suggest that the Cd^2+^ cations also play an important role in the construction of delayed fluorescent system.

In summary, for the first time, mechanochromic delayed fluorescent (MCDF) properties were achieved in double‐ligands‐based 2D metal–organic micro/nanosheets (MCDF‐1 and MCDF‐2), which show an obvious redshift emission from blue (460 or 470 nm) to green (490 nm) upon grinding at room temperature. The MCDF systems may supply new insight and enrich the typical mechanochromic fluorescence field due to the long‐lived emission. X‐ray diffraction shows the transformation from crystal to quasi‐amorphous states occurs during the MCDF process, as a result of slipping and deformation of 2D layered micro/nanosheets. Moreover, the CH_3_CN solvent can function as a self‐assembly template to achieve the reversible phase transformation between MCDF‐1 and MCDF‐2, accompanied by the recovery of the DF emission. Temperature‐dependent luminescent spectra and theoretical calculation show that the origin of reversible MCDF is the transformation between exciplex (BIM/9‐AC) and excimer (9‐AC/9‐AC), which also results in that the TADF characteristic of MCDF‐1B is more pronounced than MCDF‐1G. Therefore, the grinding stimulus could balance the energy distribution between singlet and triplet states of the 2D metal–organic micro/nanosheets effectively. It can be expected that developing 2D layered exciplex‐based hybrids with versatile and flexible weak interaction may give great promise as a new strategy to achieve MCDF materials, and thus would extend the applications in the fields of luminescent sensor, information recording and storage.

## Experimental Section

*Materials*: Analytically pure Cd(NO_3_)_2_∙4H_2_O, 9‐HAC, and BIM were purchased from the Sigma Chemical, Co. Ltd. and used without further purification.

Single‐crystal X‐ray diffraction data were collected at room temperature (293 K) on an Oxford Diffraction SuperNova area‐detector diffractometer using mirror optics monochromated Mo Kα radiation (λ = 0.71073 Å). CrysAlisPro^25^ was used for the data collection, data reduction, and empirical absorption correction. The crystal structure was solved by direct methods, using SHELXS‐2014 and least‐squares refined with SHELXL‐2014^26^ using anisotropic thermal displacement parameters for all nonhydrogen atoms. The crystallographic data for MCDF‐1 are listed in Table S1 (Supporting Information). Crystallographic data for the complex structure in this work were deposited with the Cambridge Crystallographic Data Centre (CCDC) as deposition nos. CCDC 1588834 (available free of charge, on application to the CCDC, 12 Union Rd., Cambridge CB2 1EZ, UK; e‐mail: deposit@ccdc.cam.ac.uk). PXRD analyses patterns were collected on a Rigaku Ultima‐IV automated diffraction system with Cu Kα radiation (λ = 1.5406 Å). Measurements were made in a 2θ range of 5°−50° at room temperature with a step of 0.02° (2θ) and a counting time of 0.2 s step^−1^. The operating power was 40 kV and 50 mA. The morphologies of the products were characterized by the field emission scanning electron microscopy (FESEM, S‐8010, Hitachi). Room‐temperature time‐resolved PL experiments were conducted on an Edinburgh FLS980 fluorescence spectrometer equipped with a xenon arc lamp (Xe900) and a microsecond flash‐lamp (uF900), respectively. The PL lifetimes (τ) of solid‐state samples were obtained by fitting the decay curve with a multiexponential decay function of *I*(*t*) = *A*_1_exp(−*t*/τ_1_) + *A*_2_exp(−*t*/τ_2_) + … + *A*_i_exp(−*t*/*τ_i_*), where *A_i_* and *τ_i_* represent the amplitudes and lifetimes of the individual components for multiexponential decay profiles. The time‐resolved emission decay has been measured under 1 µs pulse width and 5 Hz repetition frequency conditions. Bright‐field optical images and fluorescence microscopy images were taken from an inverted fluorescence microscope (Nikon Ti‐U), by exciting the samples with a mercury lamp. IR spectra were recorded in the range of 4000–400 cm^−1^ on a Tensor 27 OPUS (Bruker) FT‐IR spectrometer. TGA experiments were carried out on a Perkin‐Elmer Diamond SII thermal analyzer from room temperature to 500 °C with a heating rate of 10 °C min^−1^. Elemental analyses (C, H, O, and N) were performed on a Vario EL elemental analyzer.

*Electronic Structure Calculations of MCDF‐1*: All calculations were performed with the periodic DFT method using the Dmol3^27^, ^28^ module in the Material Studio software package.^29^ The initial configuration was fully optimized by the Perdew–Wang (PW91)^30^ generalized gradient approximation method with the double numerical basis sets plus polarization function (DNP). The core electrons for metals were treated by effective core potentials. The self‐consistent field converged criterion was within 1.0 × 10^−5^ hartree atom^−1^, and the converging criterion of the structure optimization was 1.0 × 10^−3^ hartree bohr^−1^. The Brillouin zone is sampled by 1 × 1 × 1 *k*‐points, and test calculations reveal that the increase of *k*‐points does not affect the results.

All details of experimental procedures are reported in the Supporting Information.

## Conflict of Interest

The authors declare no conflict of interest.

## Supporting information

Supplementary
