# Supplementary material for: Reversible Mechanochromic Delayed Fluorescence in 2D Metal–Organic Micro/Nanosheets: Switching Singlet–Triplet States through Transformation between Exciplex and Excimer
Source: Adv Sci (Weinh). 2018 Sep 27;5(11):1801187. doi: 10.1002/advs.201801187 (PMC6247076; doi:10.1002/advs.201801187)
Supplement: Supplementary file 1 — Supplementary [file ADVS-5-1801187-s001.pdf]

Copyright WILEY-VCH Verlag GmbH & Co. KGaA, 69469 Weinheim, Germany, 2018.

ADVANCED SCIENCE
Open Access

Supporting Information

for *Adv. Sci.,* DOI: 10.1002/advs.201801187

Reversible Mechanochromic Delayed Fluorescence in 2D Metal–Organic Micro/Nanosheets: Switching Singlet–Triplet States through Transformation between Exciplex and Excimer

Yongsheng Yang, Xiaogang Yang, Xiaoyu Fang, Ke-Zhi Wang, and Dongpeng Yan**

Copyright WILEY-VCH Verlag GmbH & Co. KGaA, 69469 Weinheim, Germany, 2016.

Supporting Information

Title: Reversible Mechanochromic Delayed Fluorescence in 2D Metal-organic Micro/nanosheets: Switching Singlet-Triplet States through Transformation between Exciplex and Excimer

Yongsheng Yang, Xiaogang Yang, Xiaoyu Fang, Ke-Zhi Wang, and Dongpeng Yan**

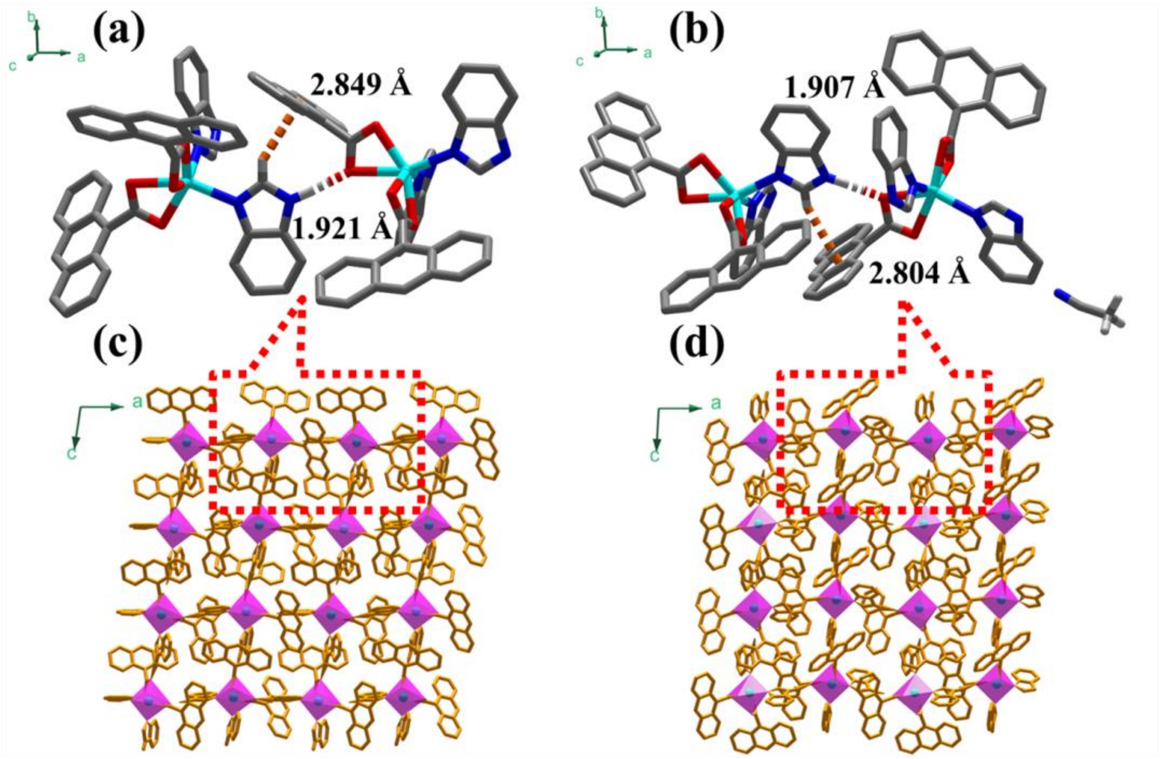

Figure S1. Coordination environments of the Cd(II) ions in MCDF-1 (a) and MCDF-2 (b). Hydrogen atoms are omitted for clarity. The 2D grid-like structures of MCDF-1 (c) and MCDF-2 (d). The

Cd coordination polyhedrons are depicted in purple, whereas the ligand links are represented by orange sticks.

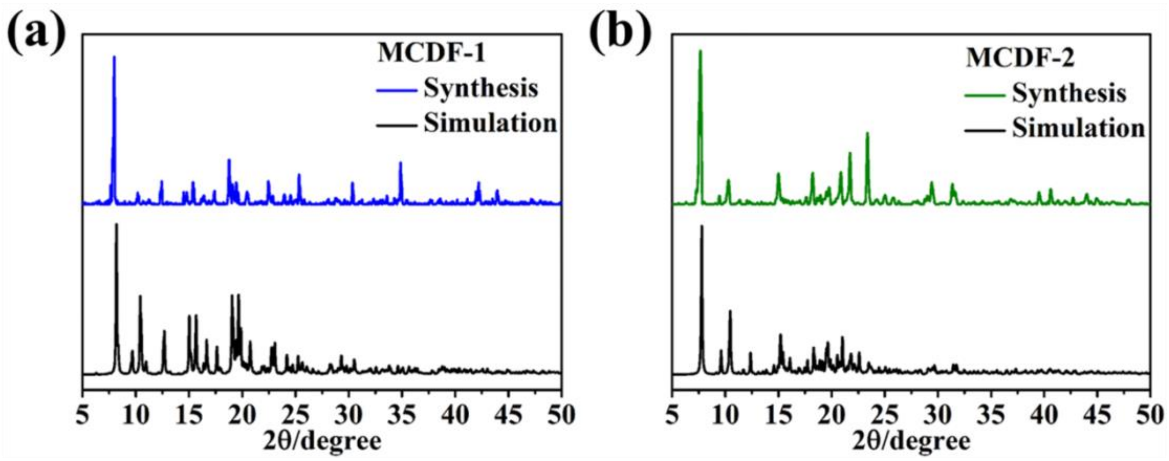

Figure S2. The calculated and experimental PXRD patterns for MCDF-1 and MCDF-2.

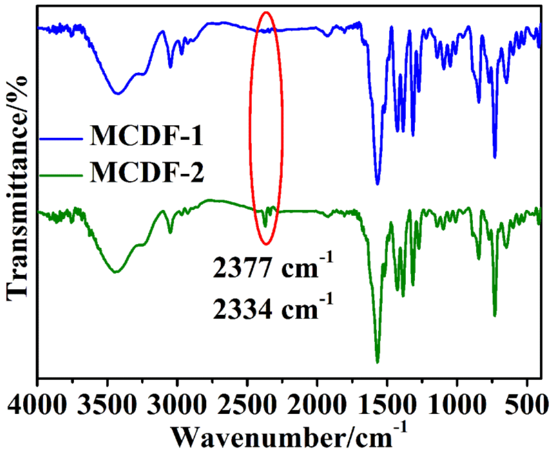

Figure S3. The FTIR spectra for MCDF-1 and MCDF-2.

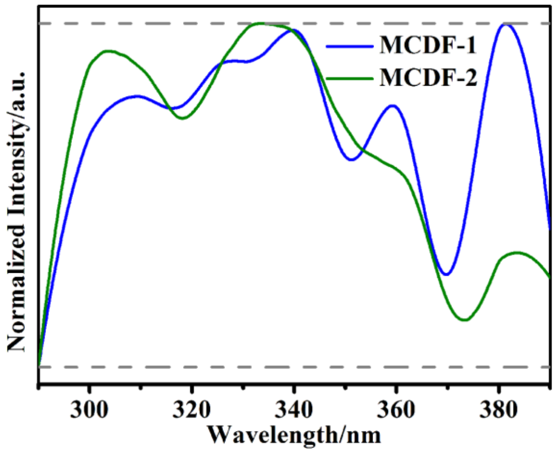

Figure S4. Normalized fluorescence excitation spectra of MCDF-1 and MCDF-2.

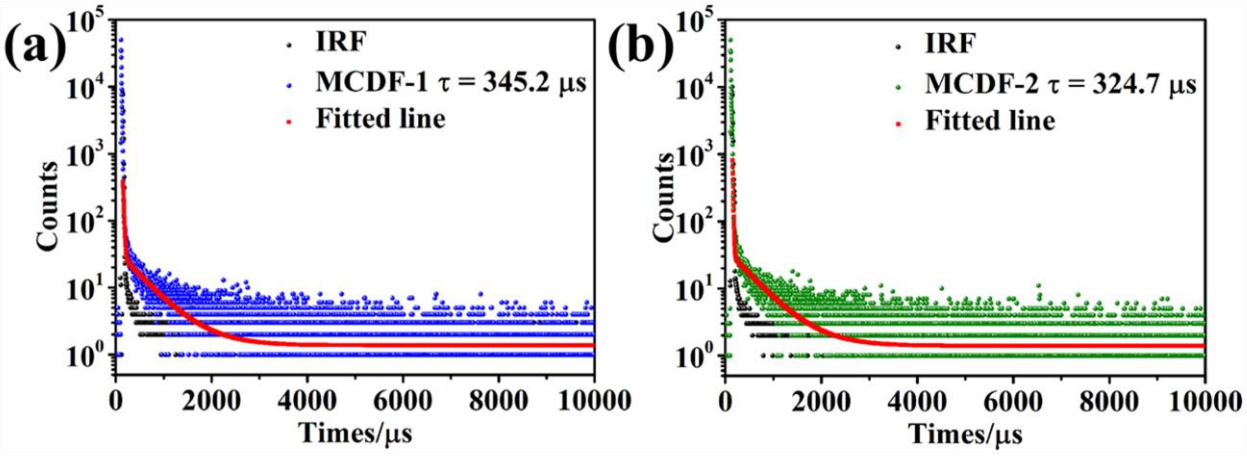

Figure S5. Time-resolved emission decay curves (the excitation wavelength is 340 nm and the emission wavelengths are 460 and 470 nm, respectively), instrument response function (IRF) and fitted lines for MCDF-1 and MCDF-2 under ambient conditions. The IRF was from the output of microsecond flash which has a pulse width of 1 μs.

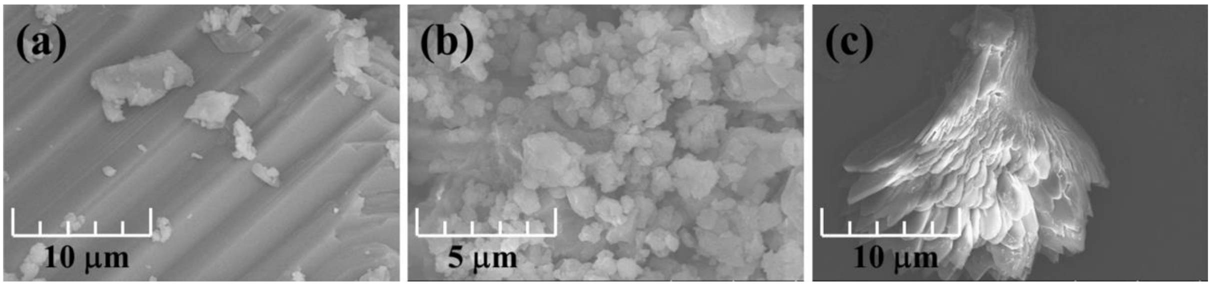

Figure S6. SEM images of MCDF-1 bulk crystal in original crystalline state (a), after grinding treatment (b) and further CH_3CN solvent treatment.

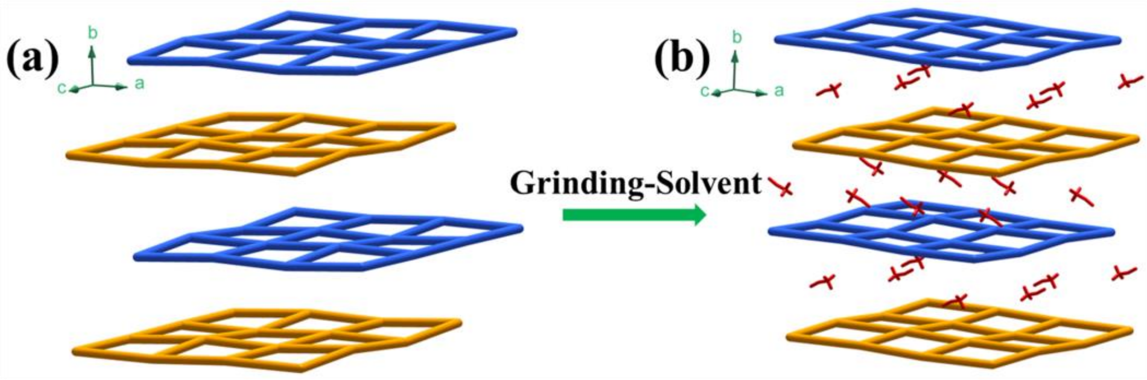

Figure S7. The schematic diagram of crystal-to-crystal transformation between MCDF-1 to MCDF-2.

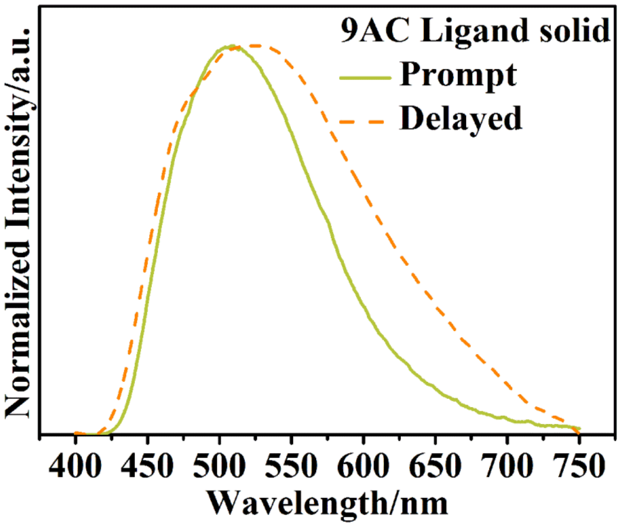

Figure S8. Normalized prompt and delay-detected photoluminescence spectra (10 μs) spectra of 9-HAC ligand in solid state.

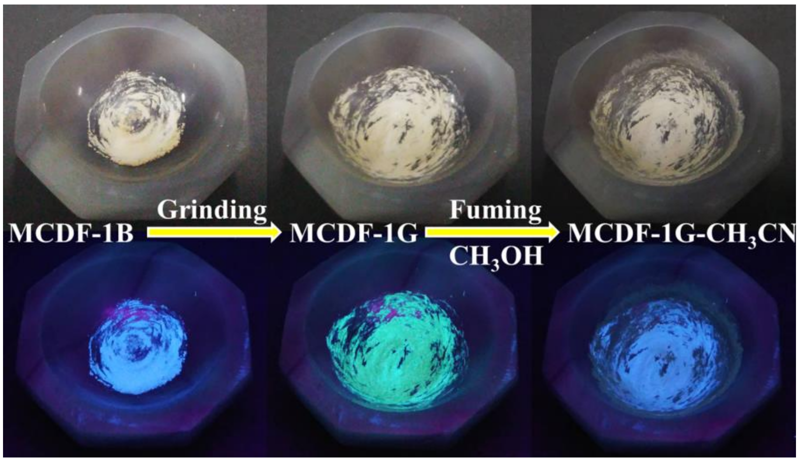

Figure S9. Solid-state emission colors of MCDF-1 upon grinding and fuming by CH_3OH under ambient light and UV lamp (irradiated at 365 nm).

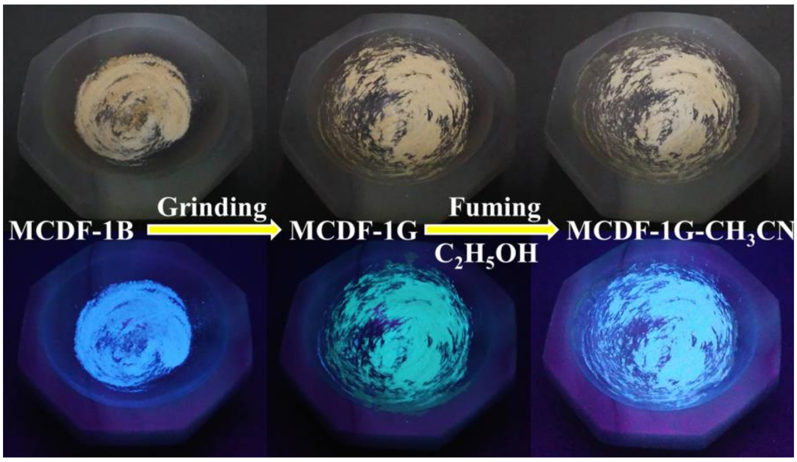

Figure S10. Solid-state emission colors of MCDF-1 upon grinding and fuming by C_2H_5OH under ambient light and UV lamp (irradiated at 365 nm).

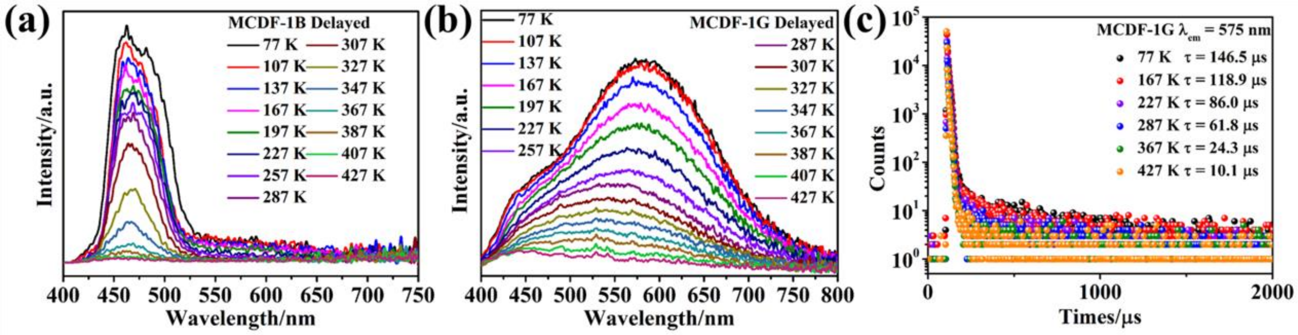

Figure S11. Delay-detected photoluminescence spectra (10 μs) of MCDF-1B (a) and MCDF-1G (b) measured at temperatures from 77 to 427 K. (c) Emission decay curves of MCDF-1G in the solid state at 575 nm with different temperatures.

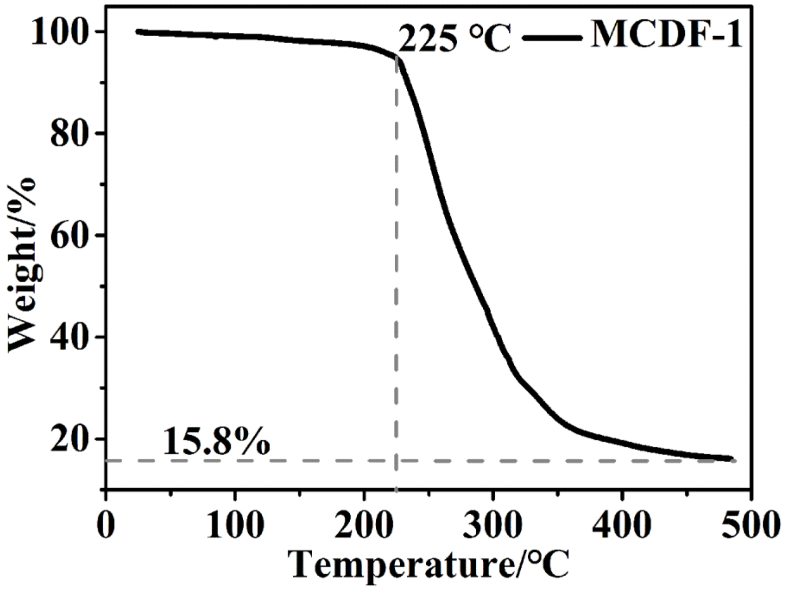

Figure S12. Thermogravimetric analysis (TGA) curve of MCDF-1.

The initial weight loss in the range from room temperature to 225 °C is related to the removal of the water and/or CH_3CN solvent molecules inside and at the surface of the sample. The BIM and 9-AC ligands in the structure gradually collapsed from 225 to 500 °C. The remaining weight for MCDF-1 is 15.8 %, which can be assigning to cadmium oxide (calculated theoretical values: 16.2 %).

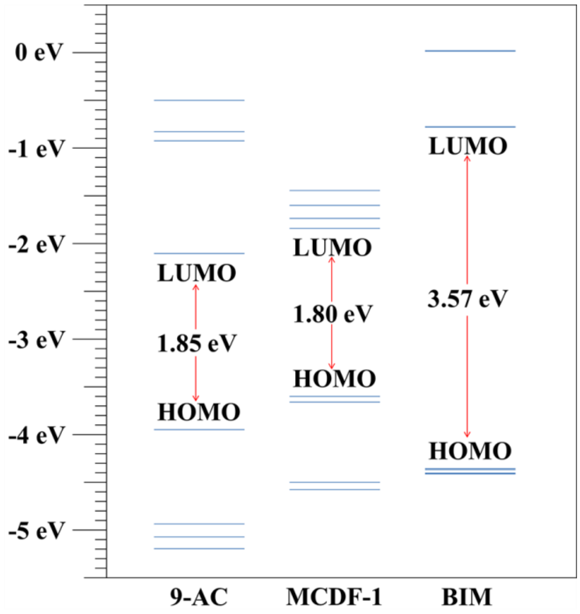

Figure S13. Energy levels of the 9-AC, BIM and MCDF-1.

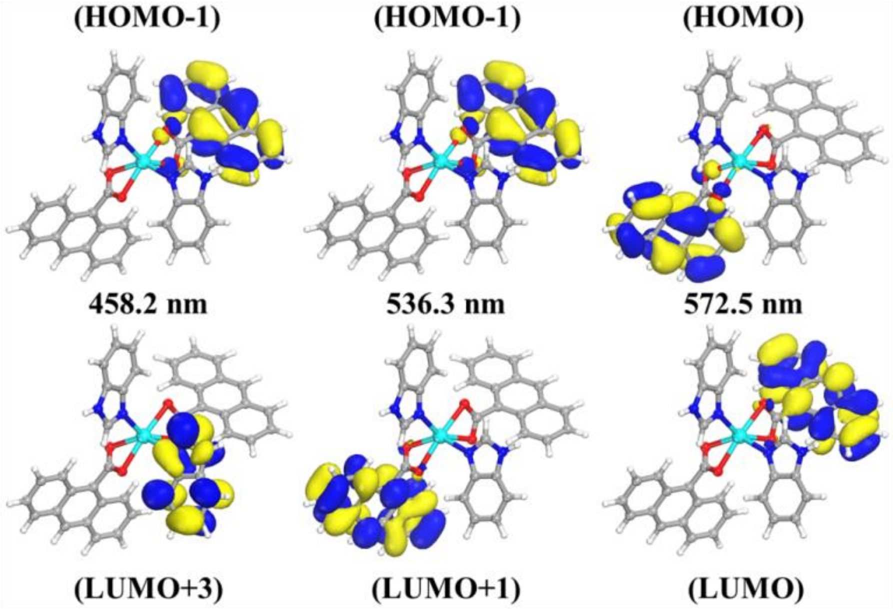

Figure S14. DFT calculations of MCDF-1: frontier molecular orbitals of MCDF-1 and corresponding transition wavelengths. HOMO and LUMO are the highest occupied molecular orbital and the lowest unoccupied orbital, respectively.

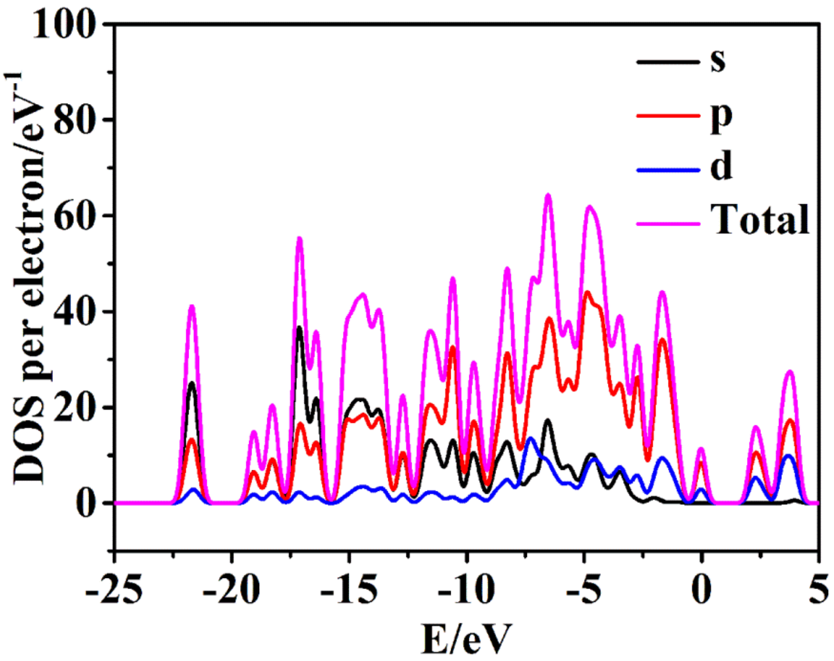

Figure S15. Total/partial electronic density of state (TDOS/PDOS) for MCDF-1.

Table S1. Crystal data and structure refinements for MCDF-1 and MCDF-2

Materials	MCDF-1	MCDF-2
Chemical formula	$C_{88}H_{60}Cd_2N_8O_8$	$C_{90}H_{62}Cd_2N_9O_8$
Formula weight	1582.33	1622.37
Crystal system	Monoclinic	Monoclinic
Space group	$P2_1$/c	$P2_1$/c
a (Å)	18.456(6)	18.230(11)
b (Å)	21.582 (10)	22.699(9)
c (Å)	18.668(7)	18.429(7)
V (Å^3)	7353.2(5)	7614.3(6)
Z	4	4
D (g cm^{-3})	1.429	1.415

μ (mm^{-1})	0.643	0.624
T (K)	293(2)	293(2)
R_{int}	0.066	0.060
Goof	1.017	1.043
$R_1(I>2\sigma(I))$	0.063	0.059
$wR_2(I>2\sigma(I))$	0.075	0.097

$R_1 = \Sigma||F_o| - |F_c||/\Sigma|F_o|$, $wR_2 = [\Sigma w(F_o^2 - F_c^2)^2/\Sigma w(F_o^2)^2]^{1/2}$

Table S3. Photophysical properties of MCDF-1B and MCDF-1G in solid state at different temperature.

		MCDF-1B		
Temperature (K)	λ_{Fl} (nm)	λ_{Ph} (nm)	S_1/T_1 (eV)	ΔE_{ST}
77	458.2	462.9	2.7062/2.6788	0.0274
107	459.2	462.3	2.7003/2.6822	0.0181
137	460.1	463.3	2.6950/2.6765	0.0185
167	460.1	461.4	2.6950/2.6875	0.0075
197	460.4	469.0	2.6933/2.6439	0.0494
227	460.4	469.9	2.6933/2.6389	0.0544
257	460.4	469.1	2.6933/2.6434	0.0499
287	459.8	469.2	2.6968/2.6428	0.0540
307	459.2	467.9	2.7003/2.6501	0.0502
327	458.9	466.7	2.7021/2.6570	0.0451

347	458.2	465.5	2.7062/2.6638	0.0424
367	456.7	465.5	2.7151/2.6638	0.0513
387	453.6	461.7	2.7337/2.6857	0.0480
407	452.3	459.8	2.7415/2.6968	0.0447
427	451.7	457.3	2.7452/2.7116	0.0336
		MCDF-1G		
Temperature (K)	λ_{Fl} (nm)	λ_{Ph} (nm)	S_1/T_1 (eV)	ΔE_{ST}
77	534.5	582.8	2.3199/2.1277	0.1922
107	537.4	582.9	2.3074/2.1273	0.1801
137	534.8	580.8	2.3186/2.1350	0.1836
167	531.9	579.7	2.3312/2.1390	0.1922
197	521.2	574.8	2.3791/2.1573	0.2218
227	511.3	570.9	2.4251/2.1720	0.2531
257	504.1	566.8	2.4598/2.1877	0.2721
287	497.8	564.8	2.4909/2.1955	0.2954
307	491.5	556.4	2.5229/2.2286	0.2943
327	488.8	554.7	2.5368/2.2354	0.3014
347	485.2	547.3	2.5556/2.2657	0.2899
367	483.3	538.7	2.5657/2.3018	0.2639
387	482.1	534.7	2.5721/2.3191	0.2530
407	475.2	528.6	2.6094/2.2348	0.2636
427	469.9	529.7	2.6389/2.2341	0.2978

Table S3. The energy levels of 9-AC, BIM and MCDF-1.

	HOMO-3	HOMO-2	HOMO-1	HOMO	LUMO	LUMO+1	LUMO+2	LUMO+3

9-AC	-5.17 eV	-5.08 eV	-4.95 eV	-3.97 eV	-2.12 eV	-0.92 eV	-0.85 eV	-0.49 eV
BIM	-4.43 eV	-4.43 eV	-4.34 eV	-4.34 eV	-0.77 eV	-0.77 eV	0.04 eV	0.04 eV
MCDF-1	-4.56 eV	-4.48 eV	-3.68 eV	-3.62 eV	-1.82 eV	-1.75 eV	-1.61 eV	-1.42 eV

Synthesis of MCDF-1: A mixture of $Cd(NO_3)_2{\cdot}4H_2O$ (0.2 mmol, 0.064 g), BIM (0.2 mmol, 0.024 g), 9-HAC (0.4 mmol, 0.088 g), CH_3CN (2 mL) and water (8 mL) was sealed in a 23 mL Teflon reactor kept under autogenous pressure at 160 ºC for 72 hours and then cooled at a speed of 10 ºC per minute to room temperature. Colorless bulk crystals were filtered off, washed with distilled water and ethanol in turn, and dried in air. Yield: 65% (based on Cd). IR (KBr, cm^{-1}): ν = 3431 (m), 3236 (m), 3047 (w), 2968 (w), 1930 (w), 1571 (vs), 1438 (s), 1385 (s), 1318 (s), 1265 (w), 1145 (w), 1099 (w), 1052 (w), 1013 (w), 847 (m), 727 (vs), 647 (m), 421 (w). Anal. calcd for MCDF-1: C, 66.74; H, 3.79; O, 8.09; N, 7.08; found: C, 66.63; H, 3.84; O, 8.23; N, 7.01.

Synthesis of MCDF-2: A mixture of $Cd(NO_3)_2{\cdot}4H_2O$ (0.2 mmol, 0.064 g), BIM (0.2 mmol, 0.024 g), 9-HAC (0.4 mmol, 0.088 g), CH_3CN (3 mL) and water (7 mL) was sealed in a 23 mL Teflon reactor kept under autogenous pressure at 150 ºC for 72 hours and then cooled at a speed of 10 ºC per minute to room temperature. Colorless bulk crystals were filtered off, washed with distilled water and ethanol in turn, and dried in air. Yield: 55% (based on Cd). IR (KBr, cm^{-1}): ν = 3433 (m), 3253 (m), 3047 (w), 2960 (w), 2377 (w), 2334 (w), 1930 (w), 1571 (vs), 1431 (s), 1391 (s), 1312 (s), 1265 (w), 1145 (w), 1099 (w), 1052 (w), 1013 (w), 847 (m), 733 (vs), 653 (m), 425 (w). Anal. calcd for MCDF-2: C, 66.57; H, 3.82; O, 7.89; N, 7.77; found: C, 66.48; H, 3.95; O, 7.93; N, 7.52.
